# Extracellular Vesicles Slow Down Aβ(1–42) Aggregation by Interfering with the Amyloid Fibril Elongation Step

**DOI:** 10.1021/acschemneuro.3c00655

**Published:** 2024-02-26

**Authors:** Vesa Halipi, Nima Sasanian, Julia Feng, Jing Hu, Quentin Lubart, David Bernson, Daniel van Leeuwen, Doryaneh Ahmadpour, Emma Sparr, Elin K. Esbjörner

**Affiliations:** † Division of Chemical Biology, Department of Life Sciences, Chalmers University of Technology, Kemivägen 10, S-412 96 Gothenburg, Sweden; ‡ Division of Physical Chemistry, Department of Chemistry, Lund University, SE-22100 Lund, Sweden

**Keywords:** amyloid-β, extracellular vesicles, EVs, amyloid kinetics, protein aggregation, Alzheimer’s disease

## Abstract

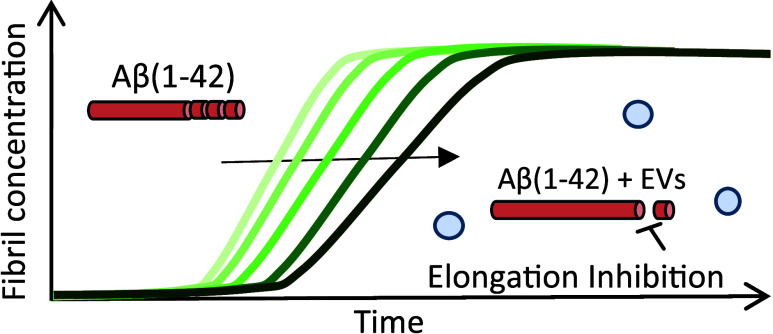

Formation of amyloid-β (Aβ) fibrils is a central pathogenic feature of Alzheimer’s disease. Cell-secreted extracellular vesicles (EVs) have been suggested as disease modulators, although their exact roles and relations to Aβ pathology remain unclear. We combined kinetics assays and biophysical analyses to explore how small (<220 nm) EVs from neuronal and non-neuronal human cell lines affected the aggregation of the disease-associated Aβ variant Aβ(1–42) into amyloid fibrils. Using thioflavin-T monitored kinetics and seeding assays, we found that EVs reduced Aβ(1–42) aggregation by inhibiting fibril elongation. Morphological analyses revealed this to result in the formation of short fibril fragments with increased thicknesses and less apparent twists. We suggest that EVs may have protective roles by reducing Aβ(1–42) amyloid loads, but also note that the formation of small amyloid fragments could be problematic from a neurotoxicity perspective. EVs may therefore have double-edged roles in the regulation of Aβ pathology in Alzheimer’s disease.

## Introduction

Alzheimer’s disease (AD) is a neurodegenerative disorder that is characterized by a progressive loss of neurons in the brain and an associated decline in memory and cognitive functions.^1^ One of the major pathological hallmarks of AD is the accumulation of extracellular senile plaques consisting of fibrillar aggregates of amyloid-β (Aβ) peptides.^1,2^ The exact causes of the formation of plaques remain unclear, and a better understanding of the molecular and cellular mechanisms that drive the underlying aberrant Aβ self-assembly and aggregation is needed. Much evidence suggests that amyloid proteins, alongside forming fibrils, also populate a variety of small and soluble oligomeric states. These have been identified as the most neurotoxic species in many cases.^3−6^ Antibodies targeting such soluble amyloid species have in fact recently reached some success in clinical trials.^7^ Furthermore, cell studies suggest that short amyloid fibril fragments, alongside various oligomers, can be neurotoxic.^8^ Thus, even though the appearance and abundance of amyloid plaques are typically not well correlated with disease severity in AD,^9^ it is still important to understand the Aβ aggregation cascade and how it can be modulated in order to provide a clearer molecular view of the pathology of the disease and thereby facilitate the identification of targets for the future much-needed development of new treatments.

Aβ peptides exist in various isoforms, of which the Aβ(1–40) variant is most abundant.^3^ The second most common and two amino acids longer Aβ(1–42) variant has been shown to aggregate more rapidly^10^ and to be the major protein component of senile plaques.^3,11^ Recent advances in the analysis of amyloid formation kinetics^12,13^ have provided detailed mechanistic insights into how the Aβ(1–42) peptide aggregates in vitro. This has pinpointed the importance of secondary nucleation (the nucleation of new aggregates on the surface of existing fibrils) as a rate-limiting step and the major source of toxic oligomeric species.^12^ Further, it has opened new avenues to understand, in molecular detail, the effects of intrinsic (mutational) modifiers^14^ as well as various external effectors^15^ and putative pharmacological agents.^16^

This study focuses on the role of extracellular vesicles (EVs) in Aβ(1–42) amyloid formation. EVs are small membrane vesicles that are secreted from most, if not all, cell types. They can, due to their small size, diffuse through biological fluids to deliver cargos such as lipids, proteins, and nucleic acids to other cells,^17,18^ even at distal sites of the body and across the blood–brain barrier.^19^ This suggests that they have functional and targeted roles in nonsynaptic intercellular communication.^20^ However, early work on EVs suggested that they also function as a means for cells to rid protein waste,^21^ which is interesting in relation to the cellular need for clearance of amyloid aggregates formed through protein misfolding and aggregation. EVs have indeed been implicated in the pathologies of many protein misfolding-related neurodegenerative diseases,^22,23^ including AD.^24−26^ Studies have reported that EVs can, in this context, have both beneficial and detrimental effects,^9^ but relatively few studies have, so far, explored their direct crosstalk with amyloid proteins.^22,24,27^ One study has shown that EVs from neuroblastoma cells accelerate the aggregation of the Parkinson-related protein α-synuclein,^22^ whereas another study showed that EVs from human pancreatic islets suppressed amyloid formation of the diabetes-associated islet amyloid polypeptide (IAPP).^28^ In AD, it is notable that a large proportion of the amyloid precursor protein (APP) cleavage events that generate Aβ peptides take place in endolysosomal compartments,^29,30^ which are conspicuously also sites for EV biogenesis. This presents an interesting and putatively pathogenic cross-section. Amyloid proteins have also been found in association with EVs, and it has therefore been suggested that EVs may be transporters in the cell–cell propagation of amyloid, which is considered important for disease progression.^31−33^ In this context, it has been reported that circulating EVs preferentially interact with prefibrillar Aβ aggregates^34^ and that EVs from AD patients contain Aβ oligomers and can transfer these to recipient neurons in cell culture.^32^ It has, on the other hand, also been reported that EVs can transport Aβ to microglia for degradation and hence contribute to clearance.^35^ These observations suggest that the cross talk between EVs and amyloid in neurodegenerative diseases may be multifaceted and with both pathological and protective outcomes.

This biophysical study focuses on the role of EVs in Aβ(1–42) amyloid fibril formation and explores their effects on the peptide’s aggregation kinetics and self-assembly mechanism, as well as on the effect of EVs on Aβ(1–42) fibril morphologies. Since EVs have been reported to be biophysically and biochemically heterogeneous,^36,37^ we compared EVs from two cell lines of different origin using human neuroblastoma SH-SY5Y as a representation of neurons and human embryonic kidney (HEK293-T) as a representation of non-neuronal cells. We used thioflavin-T (ThT) fluorescence assays to probe Aβ(1–42) aggregation kinetics in the absence and presence of the EVs, together with kinetic modeling^38^ to identify the inhibitory mechanism. The main finding of this study is that EVs from both of these cell types, potently and with surprisingly similar efficacy, reduce the rate of Aβ(1–42) fibril formation by specifically interfering with the fibril elongation step. We discuss this result in relation to observations of significant morphological changes to the resulting fibrils by atomic force and cryo-electron microscopies. Our study provides important molecular and mechanistic insights into the impact of EVs on extracellular Aβ aggregation and contributes to the understanding of their role(s) in Aβ-mediated neurodegeneration.

## Results

### Isolation and Characterization of EVs

**Figure 1:**
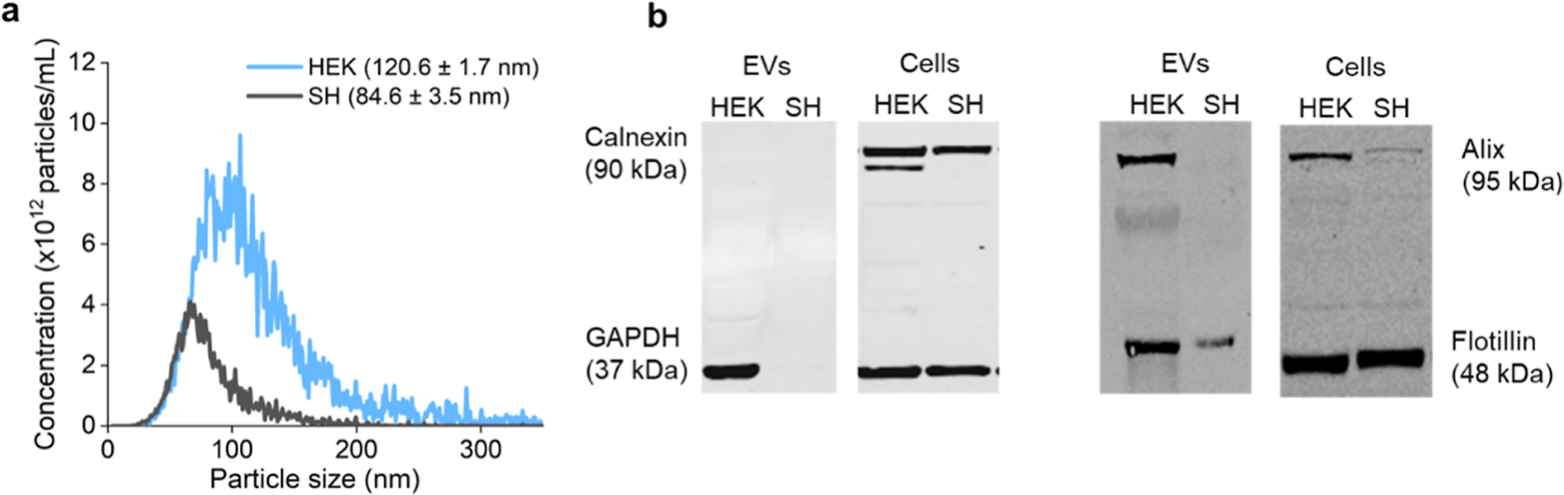
Size and molecular identity of EVs. (a) Size distributions and particle concentrations of EVs isolated from SH-SY5Y (black) and HEK293-T (blue), as determined by NTA. Mean EV diameters ± standard deviations are given in the legend. (b) Western blots showing the presence of different protein markers in the EV samples and whole cell lysates. Abbreviations: SH = SH-SY5Y and HEK = HEK293-T.

EVs from adherent cultures of SH-SY5Y neuroblastoma and HEK293-T embryonic kidney cells that had been kept under serum-free conditions were isolated by centrifugal filtration of the conditioned media,^39^ as described in the Methods section. This collects all cell-secreted EVs below the set size cutoff (220 nm, see Methods), including subtypes such as exosomes and small microvesicles. The particle size distributions and concentrations in the EV samples were determined by using nanoparticle tracking analysis (NTA) (Figure 1a, Supporting Information Table S1). The samples contained particles that were within the expected size range for exosomes (∼40 to 160 nm) and microvesicles (∼50 nm to 1 μm).^18^ We found that HEK293-T cells released more and larger (∼1.5× larger mean diameter) EVs than the SH-SY5Y cells. The protein content of the EVs was determined to be 1.65 ± 1.30 × 10^–9^ μg per particle for SH-SY5Y and 3.05 ± 0.54 × 10^–9^ μg per particle (Supporting Information Figure S1) using a BCA assay. This agrees well with other published data.^28,40^ The higher protein content in the HEK 293-T EVs can be explained with its approximately two times larger surface area (as estimated by their mean radii, Supporting Information Table S1). Western blot analysis of the EVs and of the corresponding whole cell lysates confirmed the presence of the EV-enriched protein Flotillin-1 in both EV types, but the amount was lower in SH-SY5Y EVs, despite similar abundances in the cell lysates (Figure 1b). HEK293-T, but not SH-SY5Y, EVs were also positive for EV-associated protein Alix. The lack of Alix in SH-SY5Y EVs can likely be explained by low cellular expression levels even though Alix expression in SH-SY5Y EVs was confirmed in another study.^41^ None of the EV samples contained calnexin, confirming that they were free from cellular contaminations.

### EVs Slow Down Aβ(1–42) Aggregation into Amyloid Fibrils

**Figure 2:**
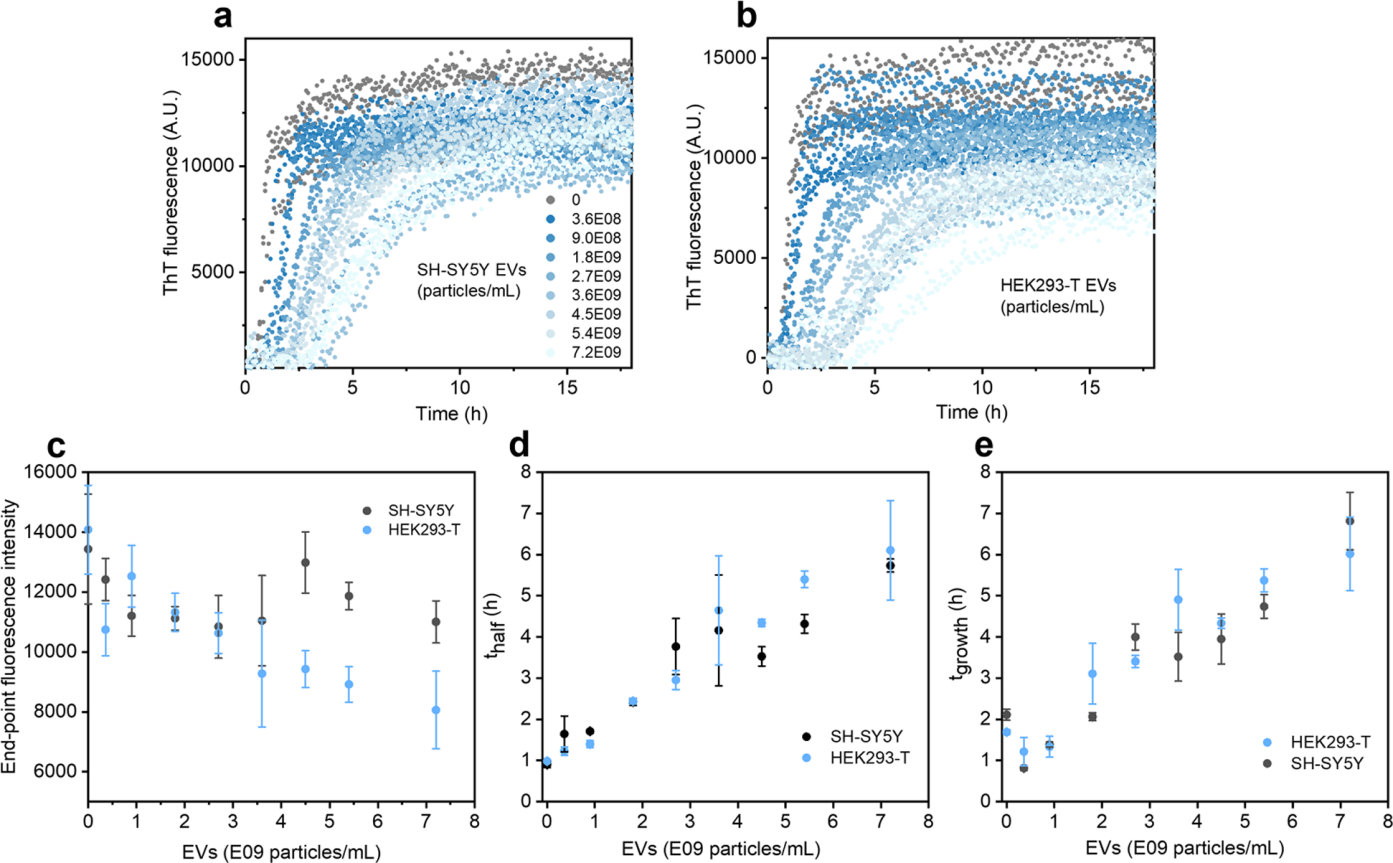
Aβ(1–42) aggregation kinetics in the presence of EVs. (a,b) Change in ThT fluorescence as a function of time, representing the aggregation kinetics of 2 μM Aβ(1–42) into amyloid fibrils in the presence of increasing concentrations of EVs purified from (a) SH-SY5Y and (b) HEK293-T cells. The EV concentrations are given in particles/mL, as indicated by the legend in (a). Three replicate kinetic curves are overlaid for each condition. (c) Change in end-point ThT fluorescence (defined as the mean ThT signal over the final 3 h of the plateau phase, which corresponds to 37 data points) as a function of increasing EV concentration. (d) Reaction half-times and (e) reaction growth-times, extracted from the data in (a,b). The error bars represent standard deviation (*n* = 3).

ThT fluorescence^42^ was used to monitor the kinetics of Aβ(1–42) amyloid fibril formation in the absence and presence of the SH-SY5Y- and HEK293-T-derived EVs. We used size exclusion chromatography-purified monomeric peptide solutions (2 μM) as the starting material to obtain reproducible kinetics.^12,43^Figure 2a,b shows the kinetic curves for Aβ(1–42) amyloid fibril formation across a range of different EV particle concentrations that are in line with reported abundances of EVs in cerebrospinal^44^ or interstitial brain fluid.^45^ The data show that both EV types slow the aggregation rate of Aβ(1–42) in a concentration-dependent manner. This was accompanied by a decrease in end-point ThT fluorescence (taken as the mean ThT signal over the final 3 h of the plateau phase for each sample, especially for the HEK293-T-derived EVs Figure 2c). SDS-PAGE analysis of the residual monomer content at the aggregation end points showed that the presence of EVs somewhat reduced the monomer conversion into fibrils (Supporting Information Figure S2). This may suggest that the EVs both slow down the aggregation kinetics and shift the monomer–fibril equilibrium or apparent solubility of Aβ(1–42), although it should be noted that we could not observe any clear trend in residual monomer concentration with increasing EV concentration. The cell culture media on its own (without EVs) had no effect on Aβ(1–42) aggregation (Supporting Information Figure S3). The change in Aβ(1–42) aggregation reaction half-times (*t*_half_) and growth-times (*t*_growth_; defined as the reaction time to increase the fibril content from 10 to 90% of the end-point maximum) as a function of EV concentration was analyzed (Figure 2d,e), which indicated an approximate sixfold reduction in the reaction rate at the highest EV concentration tested. Interestingly, there were very small differences in the aggregation modulatory effect of the SH-SY5Y- and HEK293-T-derived EVs, despite their different cellular origins, significantly different mean diameters, and differences in the abundance of at least some generic surface markers (Figure 1).

### EVs Inhibit the Elongation Step in Aβ(1–42) Fibril Formation

**Figure 3:**
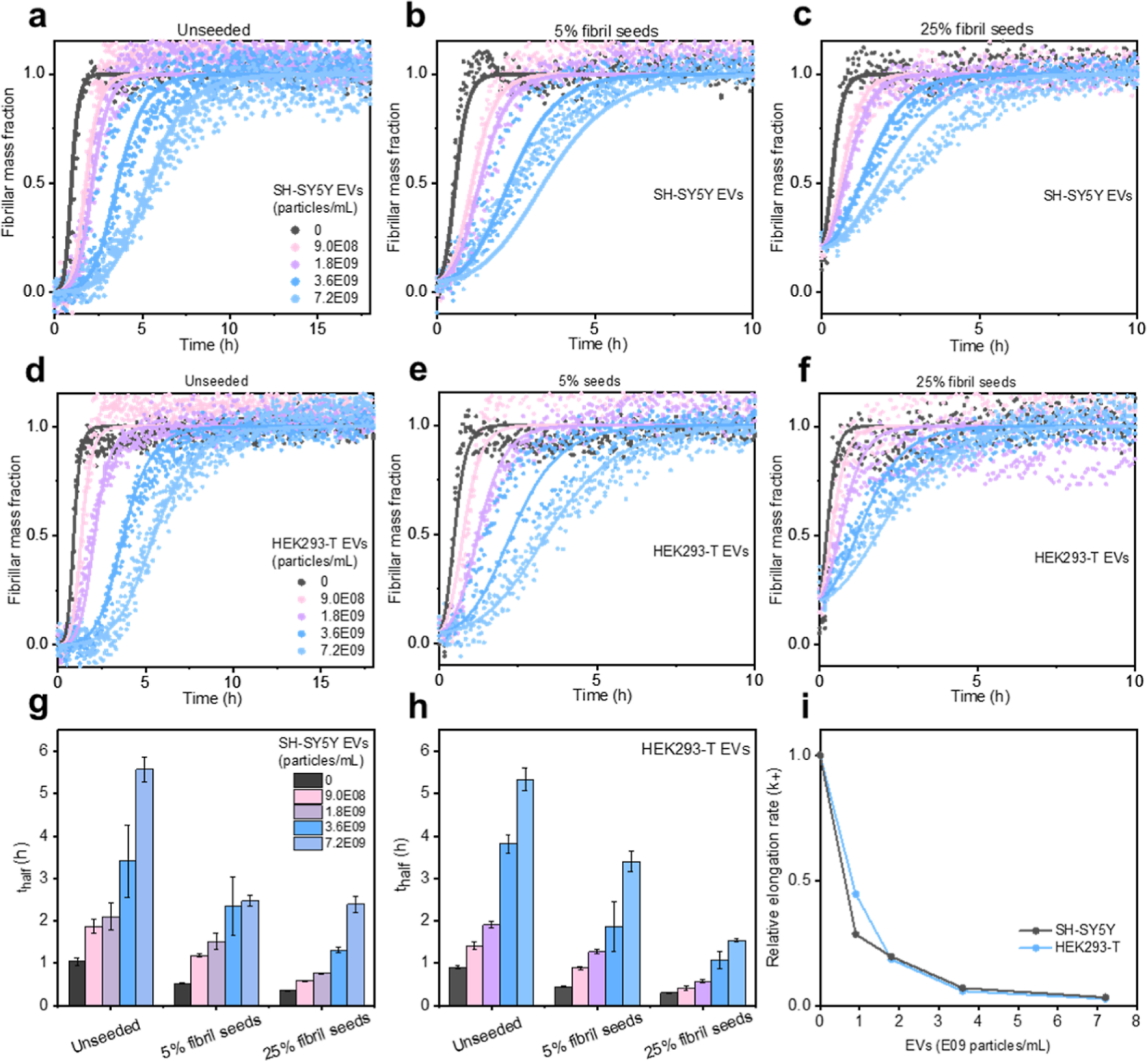
Effect of EVs on the seeded aggregation of Aβ(1–42). (a–f) Normalized Aβ(1–42) aggregation kinetic curves showing the effects of EVs in the absence (a,d) and presence (b,c and e,f) of 5 or 25% preformed Aβ(1–42) fibril seeds. Panels (a–c) and (d–f) show data for SH-SY5Y and HEK293-T EVs, respectively. The solid lines were fitted to the data using a multistep secondary nucleation model of amyloid formation setting the rate constant for elongation (*k*_+_) as a free parameter as described in the main text. The parameters underlying these fits are given in Tables S4 and S5. (g,h) Reaction half-times as a function of EV and seed concentration, derived from the data in, respectively, (a–c and d–f). The error bars represent the standard deviation (*n* = 3). (i) Change in the elongation rate constant (*k*_+_) as a function of EV concentration, as determined by the fitting of the data in a–f. The elongation rates are reported relative to that of 2 μM Aβ(1–42) aggregating in the absence of EVs.

Having determined an aggregation-reducing effect of EVs, we next used kinetic analyses and seeded aggregation experiments to pinpoint the inhibitory mechanism, which is important to explain the mode of action of a modulator, its overall efficacy, and putative downstream consequences.^16^ To first discriminate between primary nucleation and secondary mechanisms (secondary nucleation and aggregation), we repeated the aggregation kinetics experiments in Figure 2, adding different concentrations of preformed Aβ(1–42) fibrils to seed the reactions (Figure 3a–f). Seeding increased the reaction rates, which is expected as the presence of preformed fibrils bypasses the, typically slow, primary nucleation reaction step.^12,46,47^ Importantly, the inhibitory effects of the EVs remained in the presence of the seeds, as further illustrated by the half-time plots in Figure 3g,h. Previous studies have shown this to be an indication of the fact that the modulator acts on secondary reaction steps (secondary nucleation or elongation).^12,16^ Since the inhibitory effect of EVs remained even under the highest seeded conditions (25%) where elongation has been shown to dominate the amyloid formation rate,^47^ these data qualitatively suggest that EVs inhibit fibril elongation.

These observations were followed up by fitting of the kinetic data using the Amylofit web interface,^38^ as described in the Methods. We used a mathematical model that accounts for the fact that Aβ(1–42) intrinsically forms fibrils via a secondary nucleation-dominated reaction mechanism^12^ that can become saturated (i.e., lose its monomer concentration dependence) at high monomer concentrations.^48^ The model operates with two variable rate constants: the product of fibril elongation and primary nucleation (*k*_+_*k*_*n*_) and the product of fibril elongation and secondary nucleation (*k*_+_*k*_2_). We obtained a good fit to the data in Figure 3a,d with *k*_+_*k*_2_, but not with *k*_+_*k*_*n*_, as a free parameter (Supporting Information Figure S4a,b and d,e) (see Supporting Information Tables S2 and S3 for all fitted parameters and associated mean residual errors). Fitting with both *k*_+_*k*_*n*_ and *k*_+_*k*_2_ as free parameters resulted, expectedly, in the best possible fit (Supporting Information Figure S4c,f) due to the highest degree of freedom. However, only the *k*_+_*k*_2_ rate constant changed in a systematic (decreasing) manner with increasing EV concentration (Supporting Information Figure S4g,h). This supports the conclusion that EVs preferentially interfere with secondary growth processes over the primary nucleation steps.

Finally, to quantitatively determine the rate constants for primary nucleation (*k*_*n*_), elongation (*k*_+_), and secondary nucleation (*k*_2_) independently, we applied the secondary nucleation-dominated model to the seeded data in Figure 3, as described in ref (12 and 38) and the Methods section. The best fit to data was obtained with the elongation rate constant (*k*_+_) as the free parameter, as shown by the solid lines in Figure 3a–c; the other fits are shown in Supporting Information Figures S5 and S6, and all fitted rate constants are given in Supporting Information Tables S4 and S5. Taken together, all kinetic analyses, including curve shape analysis (Figure 2) and data fitting (Figure 3), support the idea that EVs inhibit the elongation step in Aβ(1–42) fibril formation. The inhibition decreases the Aβ(1–42) fibril elongation rate constants ∼30-fold for SH-SY5Y EVs and 40-fold for HEK293-T EVs at the highest EV concentrations tested (Figure 3i).

### Presence of EVs during Aggregation Alters the Size and Morphology of the Aβ(1–42) Fibrils

**Figure 4:**
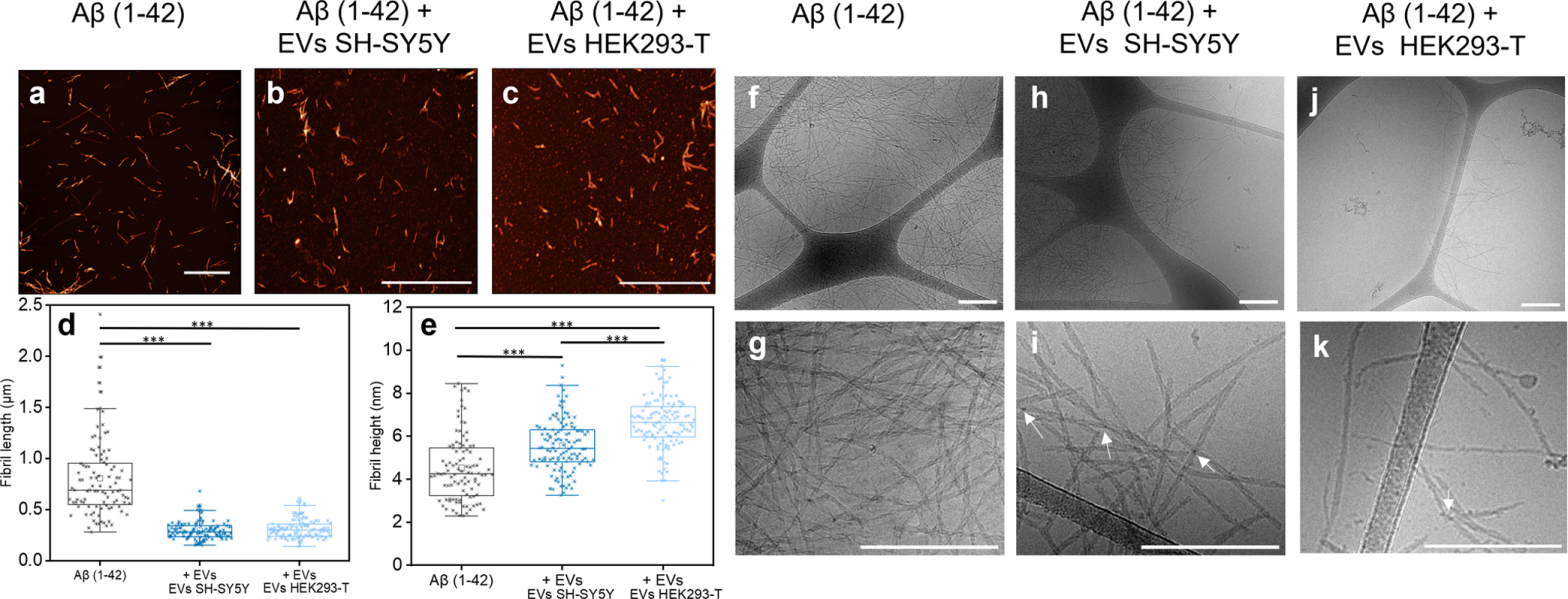
Morphological characterization of Aβ(1–42) fibrils formed in the absence and presence of EVs. (a–c) AFM images of Aβ(1–42) fibrils formed (a) in phosphate buffer with DPBS (see Methods) and (b,c) in the presence of SH-SY5Y and HEK293-T EVs. Scale bars = 2 μm. (d,e) AFM-based analysis of the distributions of (d) fibril lengths (e) and cross-sectional heights of the Aβ(1–42) fibrils formed in the absence and presence of EVs (*n* = 100–120 per condition, *** denotes *p* < 0.001 by one-way ANOVA). (f–k) Cryo-TEM images of Aβ(1–42) fibrils formed in the absence of EVs (f,g) and in the presence of EVs from, respectively, SH-SY5Y(h,i) and HEK293-T (j,k) cells. The Aβ(1–42) fibrils formed in the presence of EVs contained small dark dots, indicated by the white arrows in (i) and (k), suggestive of the dense association of EV components. Scale bars = 250 nm. All analyses have an EV concentration of 7.2 × 10^9^ particles/mL.

In addition to kinetic analysis, we examined if the presence of EVs affected the morphologies of the resulting Aβ(1–42) fibrils using atomic force microscopy (AFM) and cryogenic electron microscopy (cryo-TEM). Figure 4a–c and Supporting Information Figure S7 show representative AFM images recorded from dried samples of Aβ(1–42) fibrils taken at the end point of aggregation reactions with or without EVs (at 7.2 × 10^9^ particles/mL). The images confirm that fibrils had formed under all assayed conditions, consistent with the ThT and kinetic data (Figure 2). Analysis of the AFM images to determine fibril lengths (Figure 4d) revealed that the EVs significantly reduced average fibril lengths (from 805 ± 39 nm in the absence of EVs to 290 ± 8 and 311 ± 9 nm for SH-SY5Y and HEK293-T, respectively). The difference in the mean Aβ(1–42) fibril length in the different EV-containing samples was small and not statistically significant (one-way ANOVA, *p* = 0.37). The fragmentation of amyloid fibrils into shorter fibril length has been associated with higher toxicity.^8,49^ We did, however, only observe minor reductions in cell viability under experimental conditions relevant to the present aggregation study and no apparent differences between Aβ(1–42) fibrils formed in the absence or presence of EVs or upon treatment with EVs alone (Supporting Information Figure S8), suggesting that neither the short nor the long fibril fragments had acute cytotoxic effects at the assayed concentration.

The average heights of the Aβ(1–42) fibrils were significantly altered when formed in the presence of EVs (Figure 4e; from 4.5 ± 0.2 nm in the absence EVs to 5.6 ± 0.1 and 6.6 ± 0.1 nm for SH-SY5Y and HEK293-T EVs, respectively). This difference between fibrils formed in the presence of SH-SY5Y and HEK293-T EVs is statistically significant (one-way ANOVA, *p* < 0.001) and may relate to the fact that HEK293-T-derived EVs were larger and hence make more material available for potential co-aggregation (when supplied at the same particle concentration as used here). The Aβ(1–42) fibrils formed in the absence of EVs had an average height of 4.5 ± 0.2 nm, which is consistent with structural models of in vitro-formed Aβ(1–42) fibrils with two intertwined protofilaments obtained by solid-state NMR.^50,51^ In the case of HEK293-T-derived EVs, the increase in fibril thickness could potentially be consistent with the formation of a different fibril polymorph with three or four protofilaments,^52^ but for SH-SY5Y-derived EVs, the change is likely too small to accommodate such major rearrangement in the fibril structure. Alternatively, the change in height could result from differences in the packing of the two filaments and/or from coaggregation or coating of the Aβ(1–42) fibrils with EV components.^53^

We proceeded with carrying out cryo-TEM imaging to probe for potential fibril–EV interactions. The cryo-TEM images provided more detailed information about the morphology of the Aβ(1–42) fibrils (Figure 4k–f). The fibrils formed without EVs had, expectedly, clear filament twists (Figure 4g, Supporting Information Figure S9a–c), whereas twists were less apparent in the Aβ(1–42) fibrils formed in the presence of the EVs (Figure 4i,k, Supporting Information Figure S9d–i). The Aβ(1–42) fibrils formed in the presence of the EVs also appeared to have some small dark dots, indicating locally increased electron densities that could be related to the dense association of EV components. Notably, colocalization between intact EVs and the Aβ(1–42) fibrils was only rarely observed. An example of the colocalization of an EV to a fibril end is shown Supporting Information Figure S10. This is reasonable given that very low EV concentrations and high Aβ(1–42):EV ratios were used throughout this study due to the potency of the EV-mediated aggregation inhibition.

## Discussion

EVs have been implicated in several aspects of AD pathology, for example, as plausible candidates for systemic spreading of aggregated Aβ(1–42) peptides, as recently reviewed by Picca et al. and Jiang et al.^23,25^ Aβ peptide association with EVs has also been reported^26,54^ as well as crosstalk between EV (exosome) biogenesis and the regulation of APP processing within multivesicular body organelles.^55^ In this study, we explored another aspect of this intersection of EVs and Aβ peptides using a biophysical approach and chemical kinetics to explore a putative role of EVs as regulators in extracellular Aβ(1–42) aggregation.

The main finding of this study is that EVs, of both neuronal and non-neuronal origin, effectively slow down Aβ(1–42) aggregation by interfering with the fibril elongation step (i.e., the addition of monomers to fibril ends). This, furthermore, resulted in the formation of shorter (∼300 nm) and thicker fibrils. We observed a remarkable similarity in the effect of the two EV types, suggesting that their inhibition capacities are related to generic physical and biochemical attributes of EVs rather than to cell-type-specific EV molecular fingerprints. Our results can also be viewed in relation to the relatively scarce literature on EV-mediated effects on the aggregation of other amyloidogenic proteins. EVs have qualitatively similar effects on Aβ(1–42) and IAPP,^28^ an equally sized polypeptide but with opposite net charge at neutral pH [+2 compared to −3 for Aβ(1–42)]. The mechanism of inhibition in the IAPP case was not established, making it difficult to directly compare the results, but the opposing charges of the two peptides indicate differences in the electrostatic interactions between the peptides and the EVs. α-Synuclein aggregation has, on the other hand, been reported to accelerate in the presence of EVs.^22^ One notable difference between Aβ(1–42) and α-synuclein is the higher propensity of the latter to bind to membranes in monomeric form.^56^

We found that EVs, under the conditions and concentrations used in our work [EV concentrations of ∼10^9^ particles/mL and an initial Aβ(1–42) monomer concentration of 2 μM], caused up to ∼6-fold reductions in the overall Aβ(1–42) fibrillation rate (Figure 2d) and 30- to 40-fold decreases in fibril elongation rates (Supporting Information Tables S4 and S5). To put this in context, one may first consider EVs as pure lipid vesicles, which, according to the steadily growing literature on EV lipid compositions,^57−59^ are enriched in cholesterol and phosphatidylcholine or phosphatidylserine, as well as sphingolipids such as sphingomyelin and glycosphingolipids. Thus, even though the exact lipid composition of the SH-SY5Y- and HEK293-T-derived EVs used here have not been assessed, it is possible to make some general comparisons to the published literature. For example, we and others have found that dioleoylphosphatidylcholine (DOPC) and^60^ or dimyristoylphosphatidylcholine (DMPC)/cholesterol^61^ synthetic lipid vesicles with sizes comparable to those of EVs can catalyze Aβ(1–42) fibrillation. It has also been shown that sphingomyelin promotes Aβ(1–42) oligomerization.^62^ This suggests that synthetic lipid membranes that contain several of the most abundant EV lipid components in fact have opposite effects on Aβ(1–42) aggregation as the inhibitory effect we report with the EVs. On the other hand, a study by Sanguanini et al.^63^ suggests that phosphatidylserine may inhibit Aβ(1–42) aggregation, and we have found that monosialoganglioside 1 (GM1) also acts inhibitory (Supporting Information Figure S11). This suggests that the fine-tuned combination of lipids present in a biological vesicle may, in fact, be decisively important for their aggregation modulatory effect. However, it should also be noted that the elongation inhibitory mechanism of EVs that we report here has hitherto not been observed with lipid vesicles. Reported effects include only the modulation of primary or secondary nucleation. This may be uniquely tied to the biological complexity of the EV membrane, where variations in lipid distributions are relevant and may be important, as well as the presence of transmembrane and surface-associated EV proteins.

Importantly, there is a rather large difference in the concentrations needed to modulate Aβ(1–42) fibrillation using synthetic lipid vesicles compared to EVs. The modulatory effects observed in the abovementioned references required synthetic lipid vesicle concentrations of typically around 10^10^–10^11^ particles/ml (see Supporting Information text for calculation) to achieve smaller effects (typically no more than twofold changes to the Aβ(1–42) aggregation rate) than with EVs. Thus, although it has been recognized that the compositional complexity of lipid membranes (as existing in cell-derived EVs) is important for the outcome of lipid membrane-mediated modulation of Aβ(1–42) aggregation,^63^ our work indicates that EVs are much more potent than their synthetic, pure lipid vesicle counterparts. This, in turn, further emphasizes that EV proteins or specific lipids may be important for the inhibitory effect on Aβ(1–42). For example, EVs have been observed to contain chaperones^64^ such as Hsp70,^20^ which could potentially contribute to the inhibition of Aβ(1–42) aggregation.^65^ We report a high degree of similarity in the inhibitory effect of the SH-SY5Y- and HEK-293T-derived EVs, despite their different cellular origins and mean particle size. Ribeiro et al. reported that pancreatic EVs from healthy individuals inhibit IAPP aggregation, whereas EVs from type II diabetes patients had no effect. They speculate that observed differences in protein coverage on the EV surface (or conversely the accessibility to the lipid bilayer of the EVs) could underlie this result.^28^ We observe, on the other hand, that the protein content of the SH-SY5Y and HEK-293T differs by a factor of ∼2 (Supporting Information Figure S1), which correlates with their respective surface areas (as estimated by the difference in EV size, Supporting Information Table S1). This suggests that the SH-SY5Y- and HEK-293T-derived EVs have similar protein coverage, and this could, if one reasons along the same lines as Ribeiro et al., contribute to their similar effects on Aβ(1–42).

We concluded, using different kinetic analyses, that the EVs slow down Aβ(1–42) aggregation by primarily inhibiting the fibril elongation step. This conclusion is in agreement with the formation of shorter fibril fragments in EV-containing Aβ(1–42) samples (Figure 4). Notably, inhibition of primary or secondary nucleation should, by contrast, increase fibril lengths in a system with finite amounts of available monomer, simply because the rate of formation of new fibrils in the system is slowed down and elongation of existing fibrils is therefore favored. Fibril elongation inhibition has previously not been reported in relation to lipid-membrane-mediated amyloid modulation. It has rather been associated with end-capping molecules such as certain chaperones^66,67^ or divalent metal ions.^43,68^ The latter bind to, and induce, N-terminal loops in Aβ monomers,^69^ thus impeding their abilities to associate with fibril ends. Even though we found rare examples of intact EVs attaching to fibril ends (Supporting Information Figure S9), this appears as an energetically unfavorable interaction geometry. Moreover, based on simple geometric considerations (see Supporting Information text for calculation), and the reasonable assumption that most Aβ(1–42) monomers converted into fibrils,^70^ one can estimate the Aβ(1–42) fibril concentration in the EV-containing samples to be on the order of 10^15^ fibrils/mL at the aggregation end point. Thus, on a particle basis, the EVs exert their effects under extreme substoichiometric conditions, which appear inconsistent, on a numerical basis, with a prevalent end-capping effect. However, similar extremes in substoichiometric inhibition have been observed with linoleic acid and the amyloid-forming peptide NACore, and the effect is ascribed to the co-assembly of fatty acid aggregates and the amyloid species at the early stages of aggregation.^71^ Moreover, whereas the EV concentrations used in this work were within the physiological range,^44,45^ total Aβ(1–42) concentrations in brain fluids are generally lower^72^ even though significant concentration variation likely exists.^73^ This suggests that Aβ(1–42):EV ratios in the brain may be lower than those assessed here and that elongation inhibitory mechanisms could potentially be even more effective in vivo.

We found that the presence of EVs during the aggregation reaction increased the thickness of the Aβ(1–42) fibrils from ∼4.5 to ∼6 nm (Figure 4e) and that the thicker fibrils appeared to have a smoother surface (less apparent twist, Figure 4g,i). This could result from differences in the lateral assembly of protofilaments alone. Similar alterations to Aβ fibril morphologies have also been observed in the presence of lipid vesicles composed of POPC, GM1, and cholesterol.^74^ Furthermore, aggregation of the Parkinson’s related protein α-synuclein in the presence of phospholipid vesicles leads to the formation of lipid–protein co-aggregates with distinct morphologies compared to assemblies formed by the protein alone.^53^ It is therefore possible that Aβ(1–42), through fibril growth, can sequester lipids from the EVs. However, the larger inhibitory effect of EVs compared to that of synthetic lipid vesicles suggests that EV proteins also play an important role. It is possible that co-aggregation and coating of the fibril surface impede elongation even though ends are not specifically capped. Co-aggregated proteins and lipids may alter electrostatic and hydrophobic properties of the fibrils or induce a different fibril fold. This is important since fibril elongation mechanistically may initiate by monomer adsorption to the fibril surface and subsequent sliding of the monomer toward the fibril end,^75^ a process that could be significantly impeded by the presence of co-aggregated molecules that alter the fibril surface. Inhibition of Aβ(1–42) elongation could also, at least in part, occur indirectly due to monomer sequestration onto EVs. However, this would likely have manifested as effects on primary nucleation, as well.

A second consequence of the EV-mediated inhibition of Aβ(1–42) fibril elongation is the formation of short fibrils (∼300 nm), resulting from the skewed balance between nucleation events (formation of new aggregates) and elongation (extension of existing ones) in the reaction. This will lead to accumulation, over time, of smaller protein aggregates. Even though we could not confirm a clear difference in the toxicity of fibrils formed in the presence or absence of EVs under the conditions and low concentrations used in this study, elongation inhibition per se is associated with both amyloid toxicity^16^ and the poor efficacy of some anti-Aβ antibodies.^76^ We^8^ and others^49^ have, furthermore, shown a direct inverse correlation between the cytotoxicity of amyloid fibrils and their length, an effect that we ascribe to an increased propensity for cellular uptake,^8^ which in turn can enable higher mobility of the fibrils and promote their ability to propagate across the brain. Interestingly, the authors of a previous cell study on the role of microglia-derived EVs in neurodegeneration also reported that EVs reduce Aβ fibril length and did observe a concomitant increase in neurotoxicity.^77^

Altogether, this study explores the intersection between EVs and Aβ(1–42) peptides, focusing specifically on the biophysical effects of EVs on the process of Aβ(1–42) fibril formation. The finding that EVs, from two different cell sources, potently and equally slow Aβ(1–42) aggregation kinetics in vitro may, at first, suggest that they could confer neuroprotective effects in the context of AD pathology, especially to delay the onset and progression of Aβ aggregation in the extracellular space. However, the identification in this study of a fibril elongation inhibitory mechanism, which skews the balance between nucleation events and elongation^16^ and the demonstration that this, indeed, leads to the formation of short and potentially cell-reactive fibrils, suggests that the consequences of EV-Aβ(1–42) interactions may in fact drive neurotoxicity and contribute to the persistent accumulation of soluble Aβ(1–42) aggregates in the brain. We thus provide a possible mechanistic explanation whereby EVs co-aggregate with Aβ(1–42) fibrils in a way that renders the elongation step significantly perturbed, and shorter fibrils form. Even though more studies will be needed to pinpoint the exact interactions between Aβ(1–42) fibrils, EVs and EV proteins, and lipids, this study contributes importantly to our current understanding of Aβ pathology and on the complex balance of neurotoxic and neuroprotective roles that cell-derived EVs may have in the onset and development of neurodegenerative disorders.

## Methods

### Cell Culture and Cell Lines

SH-SY5Y cells were cultured in 1:1 medium of MEM + GlutaMAX and F-12 Nut Mix (Gibco, USA), supplemented with 10% FBS and 1% nonessential amino acids (Gibco, USA). HEK293-T cells were cultured in DMEM (Gibco, USA) supplemented with 10% FBS. Both cell lines were authenticated based on genotyping according to ANSI/ATCC standard ASN-0002 (Eurofins Genomics) and routinely verified free of mycoplasma using qPCR (Eurofins Genomics). Cells used for experiments were below passage number 20. For EV purification, cells were cultured in T75 culture flasks (Thermo Scientific) at 37 °C and 5% CO_2_ until they reached approximately 70% confluence. The cell medium was exchanged to 1:1 medium of MEM + GlutaMAX and F-12 Nut Mix without FBS (SH-SY5Y) or in serum-free opti-MEM (HEK293-T) for 48 h prior to collecting the conditioned medium (CM) for EV isolation.

### EV Isolation

CM from SH-SY5Y or HEK293-T cells, derived as described above, was filtered through a membrane with 0.22 μm pore size (VWR) followed by low-speed centrifugation (2000*g*) for 20 min to remove larger particles and cell debris. This also sets a size cutoff for EV isolation. The resulting CM was then subjected to ultrafiltration using Amicon Ultra-15 10 kDa (Millipore) spin filters at 5000*g* for 2 h, after which the collector tube was emptied and Dulbecco’s phosphate-buffered saline (DPBS) (Thermo Fisher Scientific) was added, and a second centrifugation at the same speed was performed.^39^ The EV samples in DPBS were recovered and stored at 4 °C for a maximum of 3 days for use in aggregation experiments to avoid degradation or aggregation of the EVs. EV samples for western blot were frozen and stored at −80 °C for a maximum of 1.5 months before analysis.

### Nanoparticle Tracking Analysis

EV size and concentration of all EV samples were determined by NTA using a NanoSight LM14 instrument with NTA 3.3 software (Malvern Instruments). Five 60 s videos were recorded per sample using the light scatter mode and a camera setting of 13. All samples were diluted in 0.22 μm filtered DPBS to proper concentrations prior to analysis, and the software settings were kept constant for all measurements to obtain comparable results. A detection threshold of 2 and 3 was used for EVs from SH-SY5Y and HEK293-T, respectively.

### Protein Content Analysis

The protein mass in the EV samples was quantified using the Pierce BCA Protein Assay Kit (Thermo Fisher Scientific) according to the manufacturer’s instructions. Prior to the analysis, the EV samples were lysed in 1× RIPA buffer, and the lysates were incubated for 30 min on ice and intermittently mixed by tapping the tubes. The Pierce BCA Protein Assay Kit (Thermo Fisher Scientific) was used to quantify the total amount of protein in the EV samples.

### Western Blot

EV samples were prepared as described in the EV isolation. Laemmli buffer was used to denature proteins and lyse the EV samples. All samples were boiled for 10 min with gentle shaking prior to loading onto 8–16% criterion TGX precast midi protein gels (Bio-Rad) that were run in Tris–glycine SDS buffer (1×) for 1.5 h at 120 V. Equal amounts of EV particles and cellular protein extract from both cell lines were loaded onto the gel. The precision plus protein dual color ladder (Thermo Fisher) was used for visualization of gel migration and protein size. Transfer to a 0.2 μM nitrocellulose membrane was performed for 7 min by using a Trans-Blot Turbo device (Bio-Rad). LI-COR PBS blocking buffer (LI-COR Biosciences) was used to block the membrane for 1 h under agitation and at RT. Thereafter, the membrane was incubated with primary antibodies anti-Calnexin (1:20,000, Abcam), anti-Flotillin-1 (1:1000, BD Biosciences), anti-Alix (1:1000, Cell Signaling), and anti-GAPDH (1:10,000, Abcam) overnight at 4 °C and with shaking. The primary antibodies were diluted in the blocking buffer, together with 1% Tween 20. The blots were washed three times in PBS-T before incubation with secondary antibodies goat antimouse and goat antirabbit (1:20,000, LI-COR) for 1 h and 15 min at RT and with agitation. The LI-COR Odyssey Infrared scanner was used for visualization of the bands on the membrane.

### Aβ(1–42) Expression and Purification

A plasmid encoding for Aβ(1–42) fused to the NT solubility tag^78^ was transformed into *Escherichia coli* (BL21) cells and expressed overnight. The cells were centrifuged and dissolved in 20 mM Tris–HCl, 8 M urea, pH 8.0 buffer, and frozen at −20 °C for further use. For purification of the Aβ(1–42) peptide, the bacterial cells were sonicated, centrifuged, and filtered with a 0.45 μm syringe filter and thereafter loaded onto a HisPrep FF 16/10 column (GE Healthcare) equilibrated with 20 mM Tris–HCl, 8 M urea, 15 mM imidazole, pH 8.0 buffer.^43^ The NT- Aβ(1–42) fusion protein, which carries a His-tag, was eluted in the same buffer supplemented with 300 mM imidazole and thereafter dialyzed against 20 mM Tris–HCl, pH 8.0 buffer for 2 h. This was followed by 1:20 mol equiv of TEV (tobacco etch virus) protease in the presence of 0.5 mM EDTA and 1.5 mM DTT and dialysis overnight at 4 °C to cleave off the NT tag. Next, the solution was loaded onto a HiLoad 16/600 Superdex 30 pg (GE Healthcare) size exclusion column equilibrated with a 20 mM sodium phosphate buffer, pH 8.0, and the monomeric Aβ(1–42) was eluted, aliquoted directly, and freeze-dried for storage at −20 °C for further use.

### Aβ(1–42) Aggregation Kinetics Assays

Lyophilized Aβ(1–42) was dissolved in 6 M guanidine hydrochloride and incubated on ice for 20 min. Aβ(1–42) monomers were purified just prior to each kinetics assay by size-exclusion chromatography (SEC) in 20 mM sodium phosphate buffer (pH 8.0) using a 10/300 Superdex 75 column (GE Healthcare). The Aβ(1–42) monomer concentration was determined during SEC, from the integrated area under the collected peak in the chromatogram, using an extinction coefficient of ε_280_ = 1280 M^–1^ cm^–1^. The samples for aggregation assays were prepared on ice to avoid initiating aggregation. Each sample contained 5 μM ThT (Sigma) and 2 μM Aβ(1–42) together with the indicated concentration (particles/mL) of the EVs. DPBS was added to maintain a constant salt level in all samples. [EVs were purified in DPBS, whereas the Aβ(1–42) peptide was purified without salt]. All samples were thereafter added in triplicate to nonbinding, transparent bottom, half-area 96-well microplates (Corning) and sealed with a plastic film to prevent sample evaporation. ThT emission was measured with bottom-optics using a Fluostar Optima or Fluostar Omega plate reader (BMG Labtech) and a 440 ± 10 nm band-pass filter for excitation and a 485 ± 10 nm band-pass filter for emission. All aggregation kinetic assays were performed at 37 °C and under quiescent conditions. For seeded kinetics experiments, fibril seeds prepared in the absence of EVs were collected directly from the wells of a plate used in a previous aggregation experiment and mixed with new monomer solution at indicated seed concentrations determined by the volumetric ratio of the seed and monomer solutions. The seeds were formed under the same experimental conditions as described in this section (Methods). The end-point maximum was defined as the mean ThT signal calculated from data points collected over a 3 h period in the plateau phase (corresponding to 37 data points).

### Analysis and Fitting of ThT Kinetic Curves

The acquired kinetic data were analyzed to determine the dominant mechanism of aggregation and extract rate constants. We used a secondary nucleation dominated model^12^ and the online fitting platform AmyloFit.^38^ First, the experimental data for Aβ(1–42) with no additives were fitted. This enabled the estimation of compounded rate constants for elongation and primary nucleation or elongation and secondary nucleation; *k*_+_*k*_*n*_ and *k*_+_*k*_2_, respectively, and the determination of *K*_M_ (which was kept constant to all further analysis). These values were used as initial values when analyzing Aβ(1–42) fibril formation in the presence of EVs and without fibril seeds. Thereafter, we used the same model for the seeded experiments and determined the rate constants for primary nucleation (*k*_*n*_), secondary nucleation (*k*_2_), or elongation (*k*_+_) independently, allowing one rate constant at the time to be probed as a fitting parameter while keeping the other two as global parameters, allowing them each to change with EV concentration but not with seed concentration. The goodness of fits was evaluated based on mean residual errors.

### Atomic Force Microscopy

AFM samples were prepared by depositing 10 μL samples to freshly cleaved mica surfaces that had been positively functionalized with 10 μL of (3-aminopropyl)triethoxysilane for 30 s, washed with Milli Q water, and dried with nitrogen gas. After 10 min of incubation to let the fibrils settle, the sample solutions were removed, and the mica was washed with MQ followed by drying with nitrogen gas. The ready samples were stored in closed containers until imaging was performed. AFM images were recorded using an NTEGRA system with a gold-covered crystal cantilever (NT-MDT, NSG01, force constant ∼5.1 N/m, resonance frequency ∼ 150 Hz). 256 × 256 pixel, 5 × 5 μm images were recorded for length and height calculations, and 512 × 512 pixel images (across the same sample area) were acquired for visualization purposes. All images were analyzed using Gwyddion software.^79^ Data leveling by mean plane subtraction, correction of horizontal aberrations, and minimum value shift to zero was applied to all images before manual measurement of the heights and lengths of 100–120 fibrils over 10 individual images per sample. The data were analyzed by performing a one-way ANOVA followed by statistical means comparison by two-sample *t*-test using Bonferroni’s correction [OriginPro 2020 software (OriginLab)].

### Cryogenic Electron Microscopy

Samples of Aβ(1–42) fibrils alone, EVs alone, and Aβ(1–42) fibrils formed in the presence of EVs were prepared according to the aggregation kinetics assay and EV isolation procedures described above. Small sample volumes of 4 μL were added as thin liquid films on carbon-coated copper grids, blotted with filter paper, and plunged into liquid ethane at −180 °C in an automatic plunge freezer (Leica). This freezing procedure prevents the formation of water crystals and preserves samples in their original structure. The samples were then kept in liquid N_2_ and transported in a cryoholder (Fischione model 2550) to the electron microscope (JEM 2200FS) for imaging. Zero-loss images were recorded with a TVIPS F416 camera at 200 kV acceleration voltage.

## Supporting Information Available

EV particle size determined by NTA; mean residual monomer content at the end point of aggregation; protein amount per EV particle; aggregation kinetics of medium control; fitting of ThT kinetic curves with AmyloFit and corresponding kinetic parameters; additional AFM and cryo-TEM images of Aβ(1–42) fibrils; estimation of fibril and vesicle concentrations; and cell viability assay (PDF)
