# Supplementary material for: Extracellular Vesicles Slow Down Aβ(1–42) Aggregation by Interfering with the Amyloid Fibril Elongation Step
Source: ACS Chem Neurosci. 2024 Feb 26;15(5):944–54. doi: 10.1021/acschemneuro.3c00655 (PMC10921407; doi:10.1021/acschemneuro.3c00655)
Supplement: Supplementary file 1 — cn3c00655_si_001.pdf [file cn3c00655_si_001.pdf]

SUPPLEMENTARY INFORMATION

to

Extracellular vesicles slow down Aβ(1-42) aggregation by interfering with the amyloid fibril elongation step

Vesa Halipi[1], Nima Sasanian[1], Julia Feng[1], Jing Hu[2], Quentin Lubart[1], David Bernson[1], Daniel van Leeuwen[1], Doryaneh Ahmadpour[1], Emma Sparr[2] and Elin K. Esbjörner[1,]*

[1]Division of Chemical Biology, Department of Life Sciences, Chalmers University of Technology, Kemivägen 10 S-412 96, Gothenburg, Sweden

[2]Division of Physical Chemistry, Department of Chemistry, Lund University, SE-22100, Lund, Sweden

*Corresponding author: Elin K. Esbjörner; eline@chalmers.se

1. SUPPLEMENTARY DATA (TABLES AND FIGURES)

Table S1. Mean and mode size (diameter) of EV particles collected from SH-SY5Y and HEK293-T cells as determined by NTA, corresponding to the data shown in Fig. 1a of the main text.

Sample	Size (nm)	
	Mean±SD	Mode±SD
SH-SY5Y EVs	84.9±3.5	69.2±2.4
HEK293-T EVs	120.6±1.7	92.1±1.7

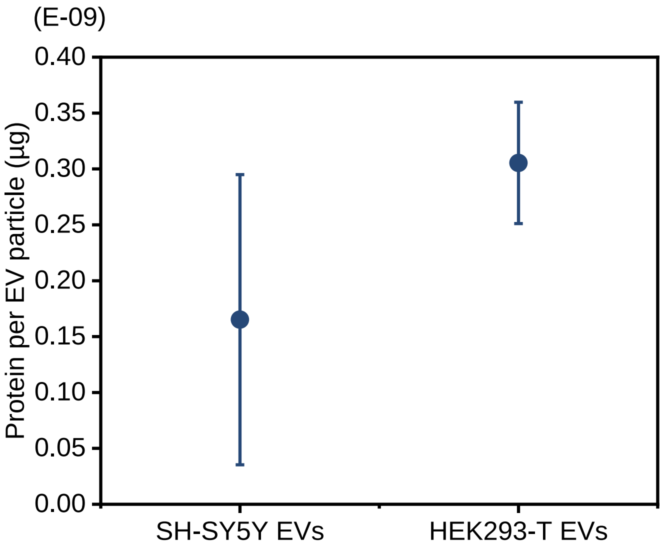

Figure S1. Quantification of protein amounts in the EVs. The total protein amount in SH-SY5Y and HEK293-T EV samples was quantified using the Pierce BCA Protein Assay Kit as described in the main text. The protein amount per EV was obtained by dividing the protein mass with the EV particle concentration in the sample as determined by NTA. The experiment was performed in biological duplicate (N=2).

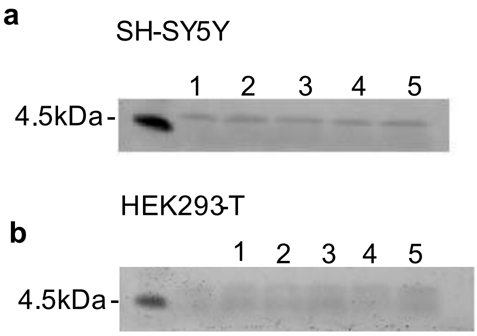

c

	SH-SY5Y	HEK293-T
**Sample:**	**Relative Quantity**	**Relative Quantity**
**1. Aβ(1-42)**	1	1
**2. Aβ(1-42) + 9.0E08 EV particles/mL**	1,36	1,06
**3. Aβ(1-42) + 1.8E09 EV particles/mL**	1,16	1,49
**4. Aβ(1-42) + 3.6E09 EV particles/mL**	0,99	1,11
**5. Aβ(1-42) + 7.2E09 EV particles/mL**	2,09	1,38

Figure S2. Residual monomer content at the aggregation end-point. SDS-PAGE was used to analyse the residual monomeric Aβ(1-42) content in the samples at the aggregation end-point. **a-b** Coomassie-stained gel showing the appearance of faint monomeric Aβ(1-42) bands at ~4.5 kDa in samples aggregated in absence and presence of increasing concentrations of **a** SH-SY5Y and **b** HEK293-T EVs. **c** Sample information and quantification of the bands as determined by relative density comparison with Image Lab (Biorad).

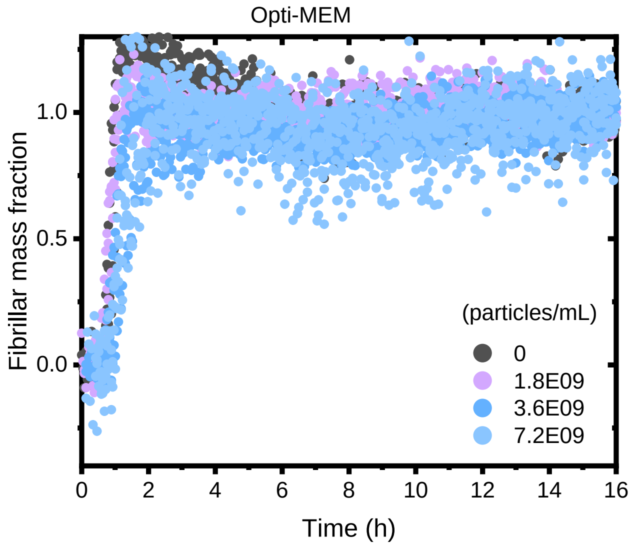

Figure S3. Aggregation of Aβ(1-42) in Opti-MEM medium control. Normalized data of 2 μM Aβ(1-42) aggregation with increasing volumes of Opti-MEM. Opti-MEM was added in volumes corresponding to an assumed EV concentration of 1.8E9 – 7.2E9 particles/mL to reflect the EV concentrations used in the study.

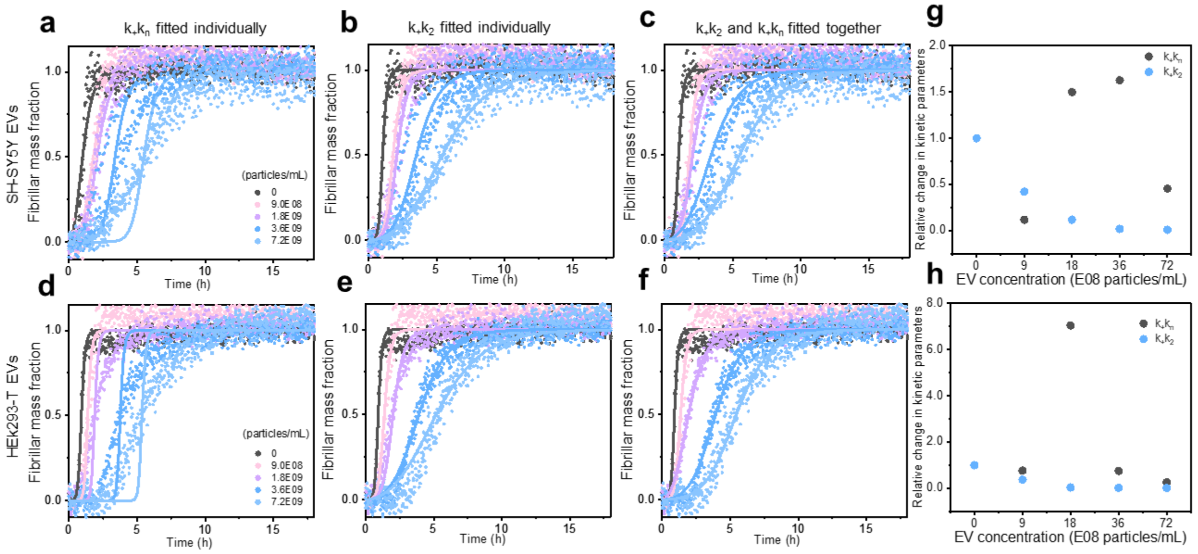

Figure S4. Kinetic modelling of experimental data using the AmyloFit web-based tool. Normalized kinetic profiles of the aggregation of 2μM Aβ(1-42) in presence of indicated concentrations of EVs, corresponding to the data shown in Fig. 3a, d in the main text. The solid lines represent fits to the data based on a saturated secondary nucleation aggregation model with different parameters set as global or fixed. **a**, **d**, k_+k_n set as free parameter (**b, e**) k_+k_2, set as free parameter, (**c,** f) k_+k_n and k_+k_2 set as free parameters. **g-h** Change in k_+k_n and k_+k_2 corresponding to the fits in (**e-f**), illustrating the random change in k_+k_n.

Table S2. Fitted kinetic parameters for Aβ(1-42) aggregation in presence of SH-SY5Y EVs (Figure S3a-c). k_+k_n is the product of the rate constants for elongation (k_+) and primary nucleation (k_n). k_+k_2 is the product of the rate constants for elongation and secondary nucleation (k_2). K_M is the Michaelis constant. A multi-step secondary nucleation dominated model was used in the web-based tool AmyloFit. K_M was determined for Aβ(1-42) aggregation with no additives and was kept as a global constant for all further analyses.

	SH-SY5Y EVs (particles/mL)					Aβ(1-42) only
	9.0e08	1.8e09	3.6e09	7.2e09	Mean residual error	
k_+k_n ($M^{-nc}h^{-2}$)	9.66e+06	1.79e+06	50.7	2.29e-09	0.0132	6.14e+09
k_+k_2 ($M^{-n2-1}h^{-2}$)	8.65e+17	5.57e+17	1.09e+17	2.00e+16	0.00627	3.93e+18
k_+k_n, k_+k_2	7.24e+08, 2.66e+18	9.2e+09, 4.65e+17	9.99e+09, 7.72e+16	2.8e+09, 3.9e+16	0.00600	6.14e+09, 3.93e+18
K_M (M^{n2})						0.0000174

Table S3. Fitted kinetic parameters for Aβ(1-42) aggregation in presence of HEK293-T EVs (Figure S3d-f). k_+k_n is the product of the rate constants for elongation (k_+) and primary nucleation (k_n). k_+k_2 is the product of the rate constants for elongation and secondary nucleation (k_2). K_M is the Michaelis constant. A multi-step secondary nucleation dominated model was used in the web-based tool AmyloFit. K_M was determined for Aβ(1-42) aggregation with no additives and was kept as a global constant for all further analyses.

	HEK293-T EVs (particles/mL)					Aβ(1-42) only
	9.0e08	1.8e09	3.6e09	7.2e09	Mean residual error	
k_+k_n ($M^{-nc}h^{-2}$)	5.94e+07	4.72e+05	0.0075	1.8e-09	0.0110	5.18e+09
k_+k_2 ($M^{-n2-1}h^{-2}$)	1.96e+18	6.63e+17	8.27e+16	2.77e+16	0.00469	5.70e+18
k_+k_n, k_+k_2	3.95e+09, 2.14e+18	3.65e+10, 1.99e+17	3.87e+09, 9.95e+16	1.37e+09, 6.48e+16	0.00406	5.18e+09, 5.70e+18
K_M (M^{n2})						0.0000556

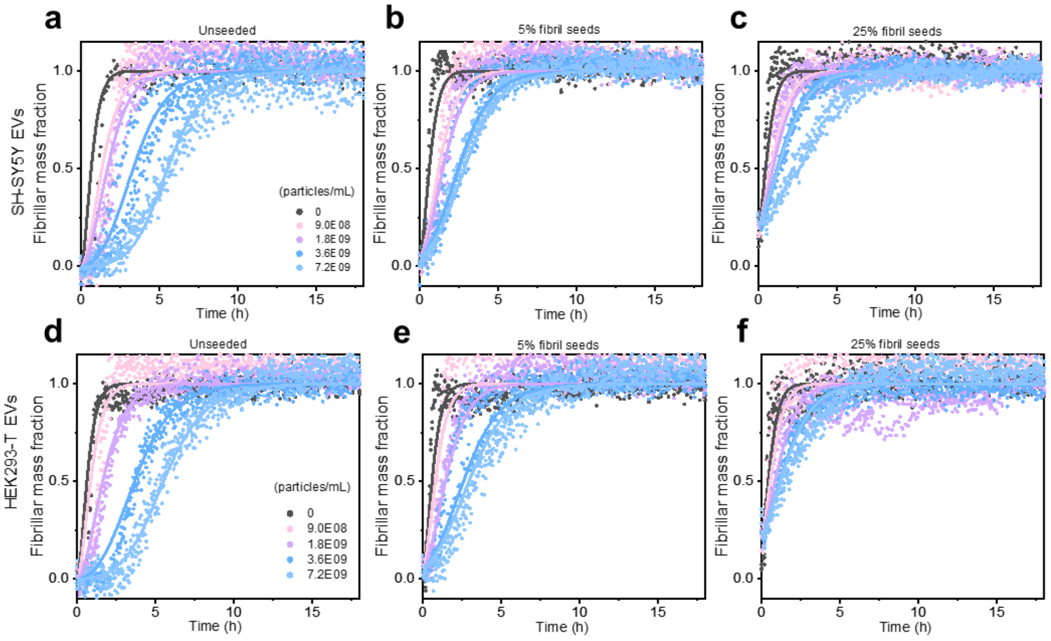

Figure S5. Modelling of experimental data using the AmyloFit web-based tool. Normalized kinetic profiles of aggregation of 2μM Aβ(1-42) in presence of SH-SY5Y EVs **a-c** or HEK293-T EVs **d-f** and increasing concentrations of pre-formed fibril seeds. Solid lines represent predictions based on kinetic modelling of reduction of the rate constant for primary nucleation, k_n. The rate constants for secondary nucleation (k_2) and elongation k_+ were kept constant.

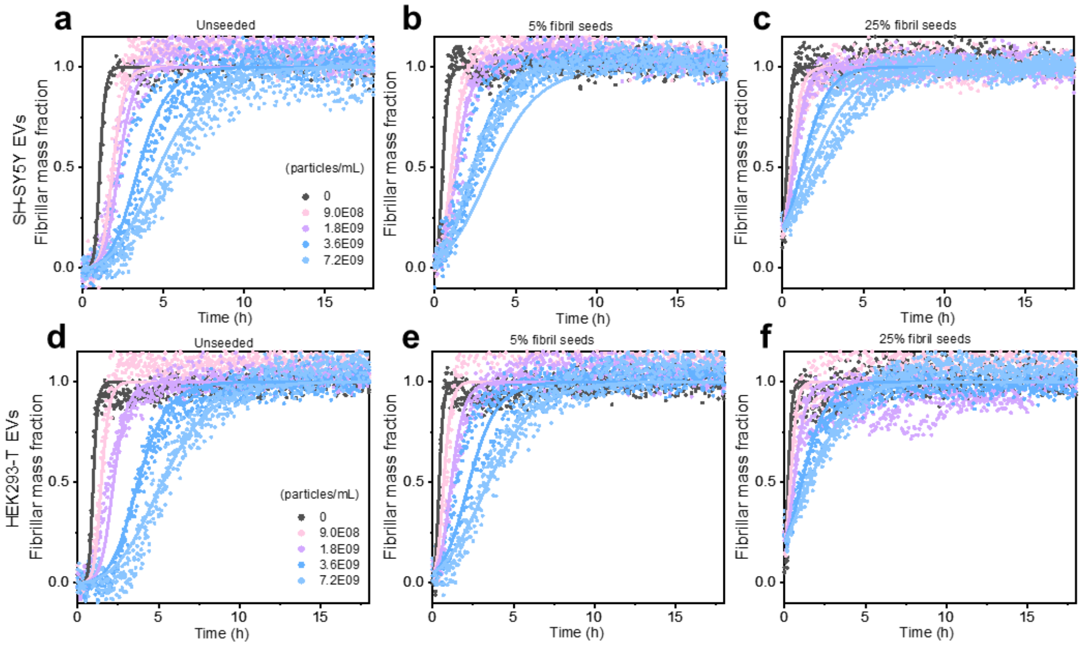

Figure S6. Modelling of experimental data using the AmyloFit web-based tool. Normalized kinetic profiles of aggregation of 2μM Aβ(1-42) in presence of SH-SY5Y EVs **a-c** or HEK293-T EVs **d-f** and increasing concentrations of pre-formed fibril seeds. Solid lines represent predictions based on kinetic modelling of reduction of the rate constant for secondary

nucleation, k_2. The rate constants for primary nucleation (k_n) and elongation (k_+) were kept constant.

Table S4. Fitted kinetic parameters for SH-SY5Y EVs. k_+ is the rate constant for elongation, k_2 is the rate constant for secondary nucleation, and k_n is the rate constant for primary nucleation.

	SH-SY5Y EVs (particles/mL)						
	0	9.0e08	1.8e09	3.6e09	7.2e09	Mean residual error	Figures
k_+ ($M^{-1}h^{-1}$)	5.09e+09	1.45e+09	9.96e+08	3.57e+08	1.64e+08	0.00489	Fig. 3a-c
k_2 ($M^{-n2}h^{-1}$)	1.84e+09	3.86e+08	2.3e+08	5.66e+07	1.82e+07	0.00500	Fig. S5a-c
k_n ($M^{-nc+1}h^{-1}$)	217	44.2	25.4	3.23	0.297	0.00654	Fig. S4a-c

Table S5. Fitted kinetic parameters for HEK293-T EVs. k_+ is the rate constant for elongation, k_2 is the rate constant for secondary nucleation, and k_n is the rate constant for primary nucleation.

	HEK293-T EVs (particles/mL)						
	0	9.0e08	1.8e09	3.6e09	7.2e09	Mean residual error	Figures
k_+ ($M^{-1}h^{-1}$)	1.78e+10	7.9e+09	3.28e+09	1.02e+09	4.71e+08	0.0074	Fig. 3d-f
k_2 ($M^{-n2}h^{-1}$)	1.75e+09	5.81e+08	1.85e+08	3.86e+07	1.23e+07	0.00751	Fig. S5d-f
k_n ($M^{-nc+1}h^{-1}$)	179	91.4	21.8	2.14	0.361	0.00903	Fig. S4d-f

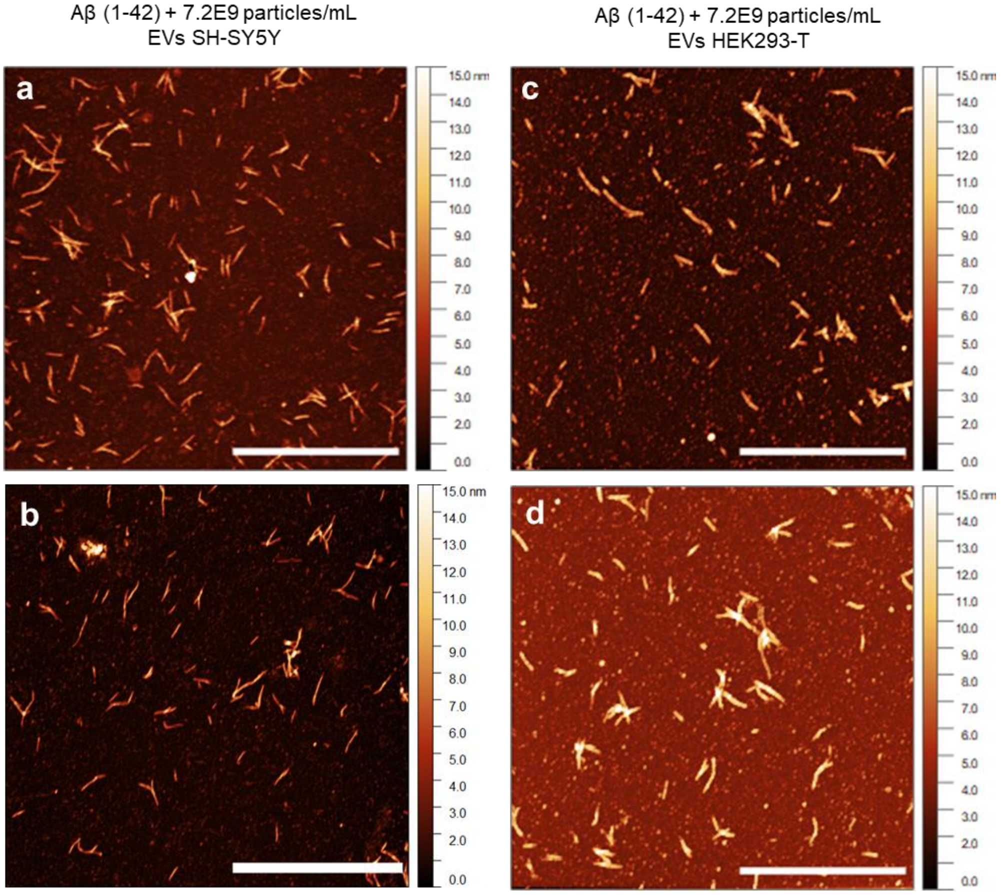

Figure S7. AFM images of Aβ(1-42) fibrils formed in in presence of EVs. a-b SH-SY5Y EVs and **c-d** HEK293-T EVs. Scale bar = 2 µm.

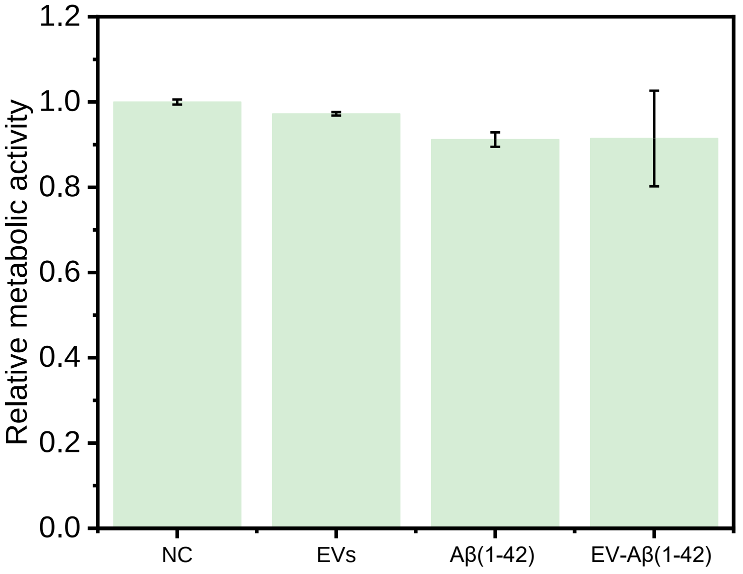

Figure S8. Effect of Aβ(1-42) fibrils on the cell viability of SH-SY5Y cells. SH-SY5Y cells were seeded at 20 000 cells/well in a 96-well plate 24 h prior to experiments. Cells were then incubated for 24 h with Aβ(1-42) fibrils formed from 2 µM monomeric Aβ(1-42) in absence or presence of EVs (7.2E09 particles/mL). The cell viability was assessed by measuring the metabolic activity using the alamarBlue™ cell viability reagent (Catalog No DAL 1100) from ThermoFisher according to the manufacturer's instructions. Error bars represent n=3 for negative control (NC, non-treated cells), EVs, and Aβ(1-42) fibrils and n=2 for EV-Aβ(1-42) fibrils.

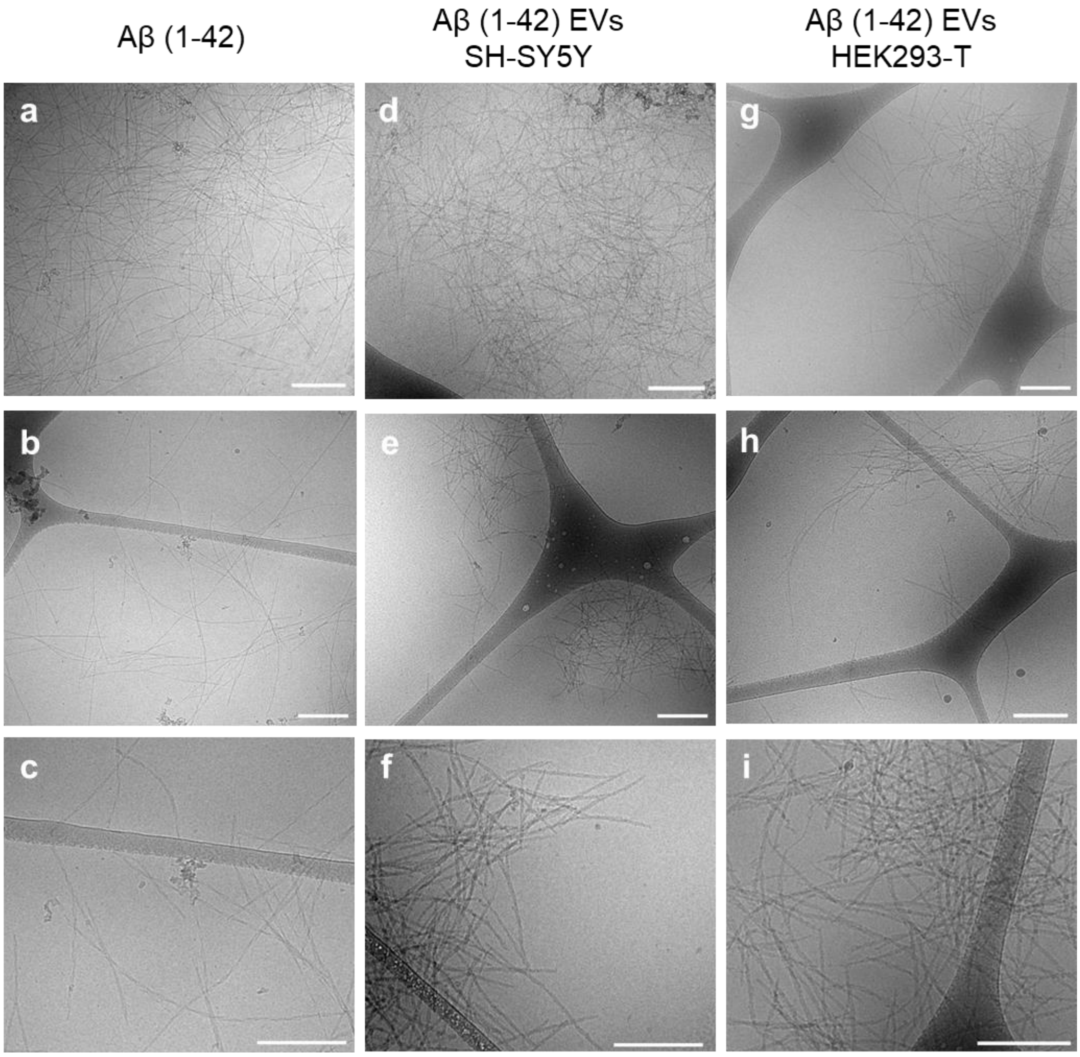

Figure S9. Cryo-TEM images of Aβ(1-42) fibrils formed in absence and presence of EVs from SH-SY5Y and HEK293-T. Cryo-TEM images of Aβ(1-42) fibrils formed in (**a-c)** absence of EVs, (**d-f)** presence of 7.2E09 particles/mL of SH-SY5Y EVs, (**g-i**) presence of 7.2E09 particles/mL of HEK293-T EVs. Scale bar = 250 nm.

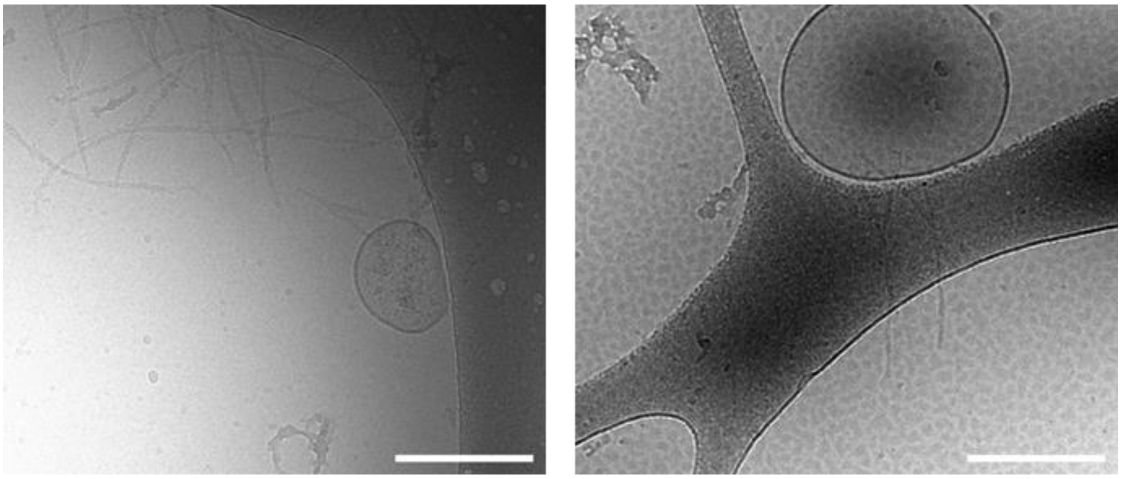

Figure S10. Cryo-TEM images of fibril-EV interactions Cryo-TEM images of EVs and fibrils after aggregation. 2E09 EVs particles/mL derived from HEK293-T cells. Scale bar = 250 nm.

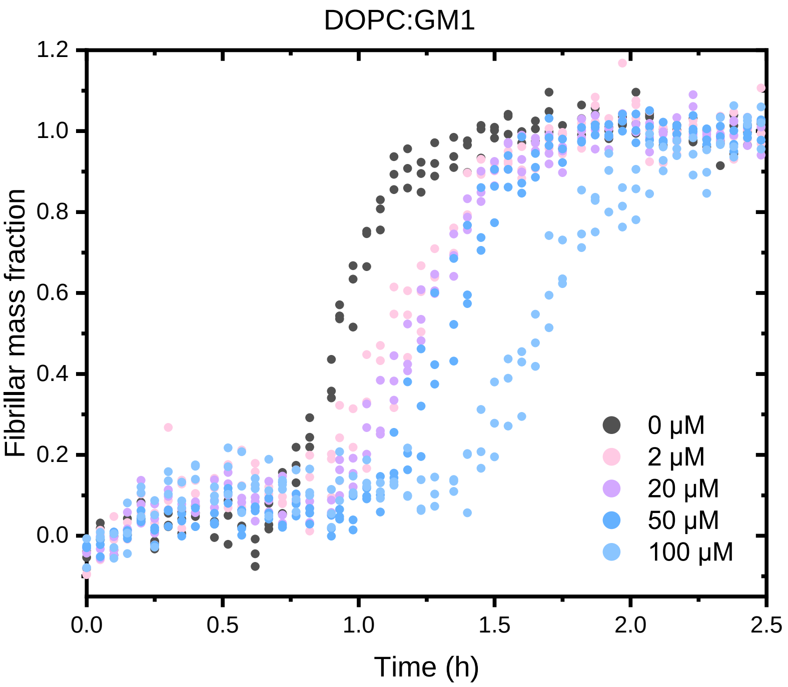

Figure S11. Aggregation of Aβ(1-42) in presence of DOPC:GM1 vesicles. Normalized data of 2 μM Aβ(1-42) aggregation with increasing concentrations of DOPC synthetic vesicles with 20% GM1.

2. SUPPLEMENTARY TEXT: Estimation of fibril and vesicle concentrations

2.1 Number of lipids in a 100 nm synthetic lipid vesicle

In the manuscript text we convert lipid concentrations into lipid vesicle (particle) concentrations for previously published data on synthetic DOPC liposomes in order to compare the amounts of synthetic lipid vesicles and EVs in the aggregating Aβ(1-42) samples.

The number of lipids in a 100 nm lipid vesicle was calculated by taking into account two leaflets, a bilayer thickness of ~5 nm[1] and an average head-group area of 70 Å^2 per DOPC lipid[2] using the following equation:

$$N_{lipids} = 4\pi r^2 + 4\pi (r - 5nm)^2 / 70\text{Å}^2$$

This results in ~ 8.1e04 lipids per vesicle.

2.2 Number of monomers in an Aβ(1-42) fibril

In the manuscript text we estimate the number of fibrils (per mL) formed in the EV-containing samples based on the following assumptions: a fibril length of 300 nm, an interstrand distance (rise) of 4.7 Å (ref), a packing of two Aβ(1-42) monomers per layer in the fibril[3, 4] and full conversion of monomers into fibrils[5].

A fibril thus contains ~1,300 monomers and a Aβ(1-42) monomer concentration of 2 μM corresponds to ~10^{15} fibrils/mL.

Author information.

Corresponding author.

Elin K. Esbjörner - Division of Chemical Biology, Department of Life Sciences, Chalmers University of Technology, Kemivägen 10, S-412 96, Gothenburg, Sweden Email: eline@chalmers.se

Authors.

Vesa Halipi - Division of Chemical Biology, Department of Life Sciences, Chalmers University of Technology, Kemivägen 10, S-412 96, Gothenburg, Sweden

Nima Sasanian - Division of Chemical Biology, Department of Life Sciences, Chalmers University of Technology, Kemivägen 10, S-412 96, Gothenburg, Sweden

Julia Feng - Division of Chemical Biology, Department of Life Sciences, Chalmers University of Technology, Kemivägen 10, S-412 96, Gothenburg, Sweden

Jing Hu – Division of Physical Chemistry, Department of Chemistry, Lund University, Box 188, SE-22100, Lund, Sweden

Quentin Lubart - Division of Chemical Biology, Department of Life Sciences, Chalmers University of Technology, Kemivägen 10, S-412 96, Gothenburg

David Bernson – Division of Chemical Biology, Department of Life Sciences, Chalmers University of Technology, Kemivägen 10, S-412 96 Gothenburg, Sweden

Daniel van Leeuwen - Division of Chemical Biology, Department of Life Sciences, Chalmers University of Technology, Kemivägen 10, S-412 96 Gothenburg, Sweden

Doryaneh Ahmadpour - Division of Chemical Biology, Department of Life Sciences, Chalmers University of Technology, Kemivägen 10, S-412 96 Gothenburg, Sweden

Emma Sparr – Division of Physical Chemistry, Department of Chemistry, Lund University, Box 188, SE-22100, Lund, Sweden

3. References

(1) Gallova, J.; Uhrikova, D.; Islamov, A.; Kuklin, A.; Balgavy, P. Effect of cholesterol on the bilayer thickness in unilamellar extruded DLPC and DOPC liposomes: SANS contrast variation study. *Gen Physiol Biophys* **2004**, *23* (1), 113-128.
(2) Lewis, B. A.; Engelman, D. M. Lipid bilayer thickness varies linearly with acyl chain length in fluid phosphatidylcholine vesicles. *J Mol Biol* **1983**, *166* (2), 211-217. DOI: 10.1016/s0022-2836(83)80007-2.
(3) Luhrs, T.; Ritter, C.; Adrian, M.; Riek-Loher, D.; Bohrmann, B.; Dobeli, H.; Schubert, D.; Riek, R. 3D structure of Alzheimer's amyloid-beta(1-42) fibrils. *Proc Natl Acad Sci U S A* **2005**, *102* (48), 17342-17347. DOI: 10.1073/pnas.0506723102.
(4) Colvin, M. T.; Silvers, R.; Frohm, B.; Su, Y.; Linse, S.; Griffin, R. G. High resolution structural characterization of Abeta42 amyloid fibrils by magic angle spinning NMR. *J Am Chem Soc* **2015**, *137* (23), 7509-7518. DOI: 10.1021/jacs.5b03997.
(5) Lindberg, D. J.; Wranne, M. S.; Gilbert Gatty, M.; Westerlund, F.; Esbjörner, E. K. Steady-state and time-resolved Thioflavin-T fluorescence can report on morphological differences in amyloid fibrils formed by Abeta(1-40) and Abeta(1-42). *Biochem Biophys Res Commun* **2015**, *458* (2), 418-423. DOI: 10.1016/j.bbrc.2015.01.132.
